# Accuracy of combined multi-parametric MRI and PSMA PET-CT in diagnosing localized prostate cancer: newer horizons for a biopsy-free pathway

**DOI:** 10.1186/s41824-023-00182-5

**Published:** 2023-11-10

**Authors:** Aditya Prakash Sharma, Rajender Kumar, Rohit Chauhan, Shiraz Akif Ziauddin, Shanky Singh, Harmandeep Singh, Sudheer Kumar Devana, Ujjwal Gorsi, Girdhar Singh Bora, Ravimohan S. Mavuduru, Santosh Kumar, Uttam K. Mete, Bhagwant Rai Mittal

**Affiliations:** 1 Department of Urology, Advance Urology Centre, PGIMER, Nehru Hospital, 160012 Chandigarh, India; 2 Department of Nuclear Medicine, PGIMER, Chandigarh, India; 3 Department of Radiodiagnosis, PGIMER, Chandigarh, India

**Keywords:** mpMRI, PSMA PET, Prostate cancer, SUVmax

## Abstract

**Introduction:**

Prostate-specific antigen (PSA) is a reliable biomarker for identification of prostate cancer, although a biopsy is still the gold standard for detecting prostate cancer. Similar to higher PIRADS lesions on MRI, the maximal standard uptake value (SUV max) on PSMA PET is linked to a higher likelihood of prostate cancer. Can an mpMRI in conjunction with PSMA PET Scan accurately predict prostate cancer and further trigger omission of biopsy similar to other solid organ urological malignancies?

**Methods:**

Ga-68 PSMA PET and mpMRI were performed for each patient who was a part of this retrospective study. The PET-positive lesion's maximum standardized uptake value (SUVmax) was recorded. Prostate biopsies were performed on patients who had PSMA PET avid lesions and a PIRADS score of 4 or 5. Robot-assisted radical prostatectomy (RARP) was afterward performed on patients who had cancer on their prostate biopsy. The prostatectomy specimen's histopathological information was recorded. Cutoff values and correlations between the variables were determined using the ROC curves and Pearson's correlation test.

**Result:**

On the basis of suspicious DRE findings or elevated PSA, 70 men underwent mpMRI and PET scans. PIRADS 4 patients had a median (IQR) SUVmax of 8.75 (11.95); whereas, PIRADS 5 patients had an SUVmax of 24.5 (22). The mean SUVmax for patients whose biopsies revealed no cancer was 6.25 ± 1.41. With an AUC of 0.876 on the ROC curve, it was found that there was a significant positive correlation between the results of the mpMRI and PET scans and those of the histopathological investigation. A SUVmax ≥ 8.25 on PSMA PET for a PIRADS 4/5 lesion on mpMRI will aid in correctly predicting malignancy, with a sensitivity of 82.8% and specificity of 100%.

**Conclusion:**

The findings of this study were positive and indicated that patients with a high suspicion of prostate cancer on mpMRI and PSMA PET (PIRADS ≥ 4 and SUVmax ≥ 8.25). This study substantiates the fact that a combination of mpMRI and PSMA PET can accurately predict localized prostate cancer.

## Introduction

Prostate cancer is one of the most common malignancies among men, and a global increase in its incidence is estimated by some reports on account of an increase in life expectancy (Rawla 2019). Despite having a reasonable biomarker in the form of Prostate-specific antigen (PSA) for its detection, biopsy remains the gold standard for diagnosing prostate cancer. Multi-parametric Magnetic resonance imaging (mpMRI) has been instrumental in the increased detection of clinically significant prostate cancer. However, it is limited by its low positive predictive value, which ranges from 34 to 68% (Ghai and Haider 2015; Ahmed et al. 2017). Hence, there is still no imaging or other means by which we can accurately pinpoint prostate cancer. It is in stark contrast to many other solid organ malignancies, such as renal cell carcinomas, where a surgical plan can be carried out based on imaging. Even for a malignancy such as pancreatic cancer where a moribund procedure such as Whipple procedure is performed without biopsy based solely upon the imaging findings (Knipper et al. 2023).

The Ga-68 PSMA PET-CT scan has established itself as imaging modality for advanced prostate cancer (Hofman et al. 2020). The maximum Standardized uptake value (SUV max) is associated with a higher probability of malignancy similar to higher PIRADS lesions seen on MRI (Tsechelidis and Vrachimis 2022 Jan). Whether a combination of MRI with PSMA PET scan suggests a higher probability of prostate cancer has been a topic of debate (Pepe et al. 2022; Caracciolo et al. 2022; Duan et al. 2023). PRIMARY study established that a combination of MRI and prostate-specific membrane antigen positron emission tomography reduces false negatives for clinically significant prostate cancer (csPCa) compared with MRI alone hence reducing the number of prostate biopsies required to diagnose cancer prostate (Emmett et al. 2021). In a study reported from our center we have shown that PSMA PET targeted limited core biopsy is accurate in diagnosing localized prostate cancer (Kumar et al. 2022). With this background we conducted this study to see if a combination of these two imaging techniques (mpMRI and PSMA PET-CT) can aid in diagnosing prostate cancer and how accurate a combination of mpMRI and PSMA PET scan is in diagnosing prostate cancer. Also, we wished to see the association of PIRADS score, SUVmax and Gleason grade for patients with localized prostate cancer.

## Material and methods

This retrospective analysis of prospectively maintained data of patients was accessed and the study was conducted from January 2021 to January 2023 at our tertiary care center with the approval of the Institutional Review Board (INT/IEC/2023/SPL-489). All patients suspected of prostate cancer based on elevated PSA values (PSA > 4 ng/ml), findings of digital rectal examination, and having localized disease on imaging were included in the study. Patients with positive nodes on PET scan, locally advanced features such as extraprostatic extension, seminal vesicle invasion, metastatic disease, and active urinary tract infection were excluded from the study.

Ga-68 PSMA PET and mpMRI were used as the imaging modalities for all patients included in the study. MRI was done using 3 Tesla scanners (MAGNETOM Verio;Siemens healthcare) and reported by an expert radiologist (U.G.) (with over 10 years of experience in reading MRI) using the PIRADS v2 system. Whole-body (vertex to midthigh) PET/CT was performed by using a dedicated PET/CT scanner (Discovery MIDR; GE Healthcare Systems) 40–60 min after intravenous ad- ministration of 111–185 MBq of 68 Ga PSMA [Glu-NH- CO–NH-Lys (Ahx)-HBED-CC]. PET was performed in six to eight bed positions (2 min per bed position). Intravenous contrast (300 mg/mL, 100 mL at a rate of 3 mL/second; Iohexol, Omnipaque) was administered and CT imaging began 70 s after contrast agent injection. The acquisition parameters for CT were as follows: 120 kVp; smart mA (100–350 mA); rotation time, 0.5 s; and pitch, 0.984:1. PET image reconstruction was performed with iterative reconstruction (Vue Point FX, Ordered Subset Expectation Maximization) with the following parameters: 24 subsets; two iterations; 192 × 192 matrix; and Gaussian filter, 5.5 mm full width at half maximum. CT images were reconstructed by using a 512 × 512 matrix and 1.25-mm section thickness. A distinct focal area of PSMA uptake above background within the prostate was considered to be a positive result at PET (hereafter, PET-positive ions), whereas lack of or inhomogeneous PSMA uptake was a negative result at PET. Two nuclear medicine physicians (R.K. and B.R.M., with 10 years of experience in PSMA PET and the center caters to about 1200 PSMA PET Scans annually) interpreted the scans in consensus. The focal PSMA expression in the prostate was interpreted as PET-positive, while no uptake or inhomogeneous tracer uptake in the prostate was interpreted as PET-negative. The maximum standardized uptake value (SUV_max_) of the PET-positive lesion was noted. Patients with a PIRAD score of 4 or 5 and PSMA PET avid lesions were subjected to a targeted with or without systematic prostate biopsy. The time gap between the MpMRI and PSMA PET-CT was about 7–10 days.

**Fig. 1:**
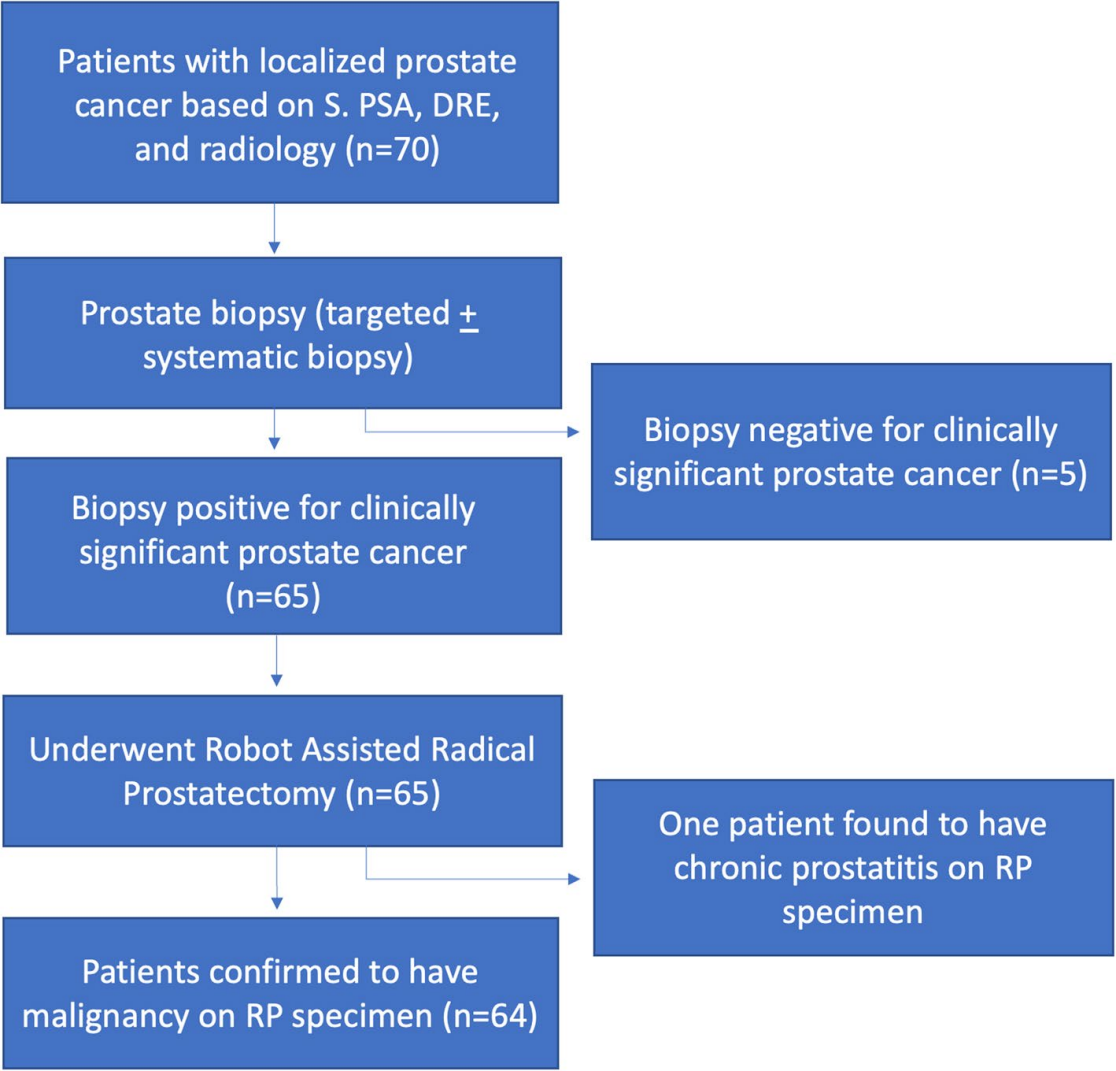
Flowchart depicting the selection and drop out criteria

Patients with malignancy on prostate biopsy later underwent Robot-Assisted Radical Prostatectomy (RARP) using the Da Vinci Si Robot-Assisted Surgical System (Intuitive Surgical, Sunnyvale, California, United States of America). All patients with Gleason grade group 1 disease were offered active surveillance; however, those who opted for RARP over active surveillance were included in the study. The histopathology data from the prostatectomy specimen were noted. Figure 1 shows the selection and drop out criteria of patients in this study. All the data were recorded in an excel sheet, and analysis was done using SPSS v24.0. Measures of central tendencies, such as mean and median, and dispersion measures, such as standard deviation, were used for descriptive statistics. Kolmogorov–Smirnov’s normality test was performed for all variables. Variables without normal distribution were expressed as median and IQR. Pearson’s correlation test and ROC curves were used to establish cutoff values and relationships between the variables.

## Results

Seventy men underwent mpMRI and PET scans based on suspicious DRE findings or raised PSA. The mean age of the patients at presentation was 66 years ± 7.5 years. Patients having localized prostate cancer on clinical evaluation were included. Table 1 depicts the patient characteristics included in our study. Thirty-eight patients had a PIRADS score of 4. The remaining thirty-two had a PIRADS score of 5.

**Table 1:** Patient characteristics

Variable	Mean (± SD) or n (%)
Age at presentation (year)	66 (± 7.5)
PSA (ng/ml; n = 70)	8.1 (± 0.8)
*Clinical T stage*	
T1c	42 (60%)
T2a	21 (30%)
T2b	5 (7%)
T2c	2 (3%)
*PIRADS*	
4	38 (54%)
5	32 (46%)
SUV max (n = 70)	16.75 (21.72)
*ISUP grade group (prostatectomy specimen)*	
No cancer	6 (8.5%)
1	17 (24.2%)
2	31 (44.3%)
3	10 (14.3%)
4	3 (4.3%)
5	3 (4.3%)

ISUP = International Society of Urological Pathology; MRI = magnetic resonance imaging; PET = positron emission tomography; PIRADS = Prostate Imaging Reporting and Data System; PSA = prostate-specific antigen; SUVmax = maximum standardized uptake value

The median (IQR) SUV_max_ of patients who underwent PSMA PET scans was 16.75 (21.72). The median (IQR) SUV_max_ of patients with a PIRADS 4 score on MRI was 8.75 (11.95), while those with PIRADS 5 were 24.5 (22).

**Fig. 2:**
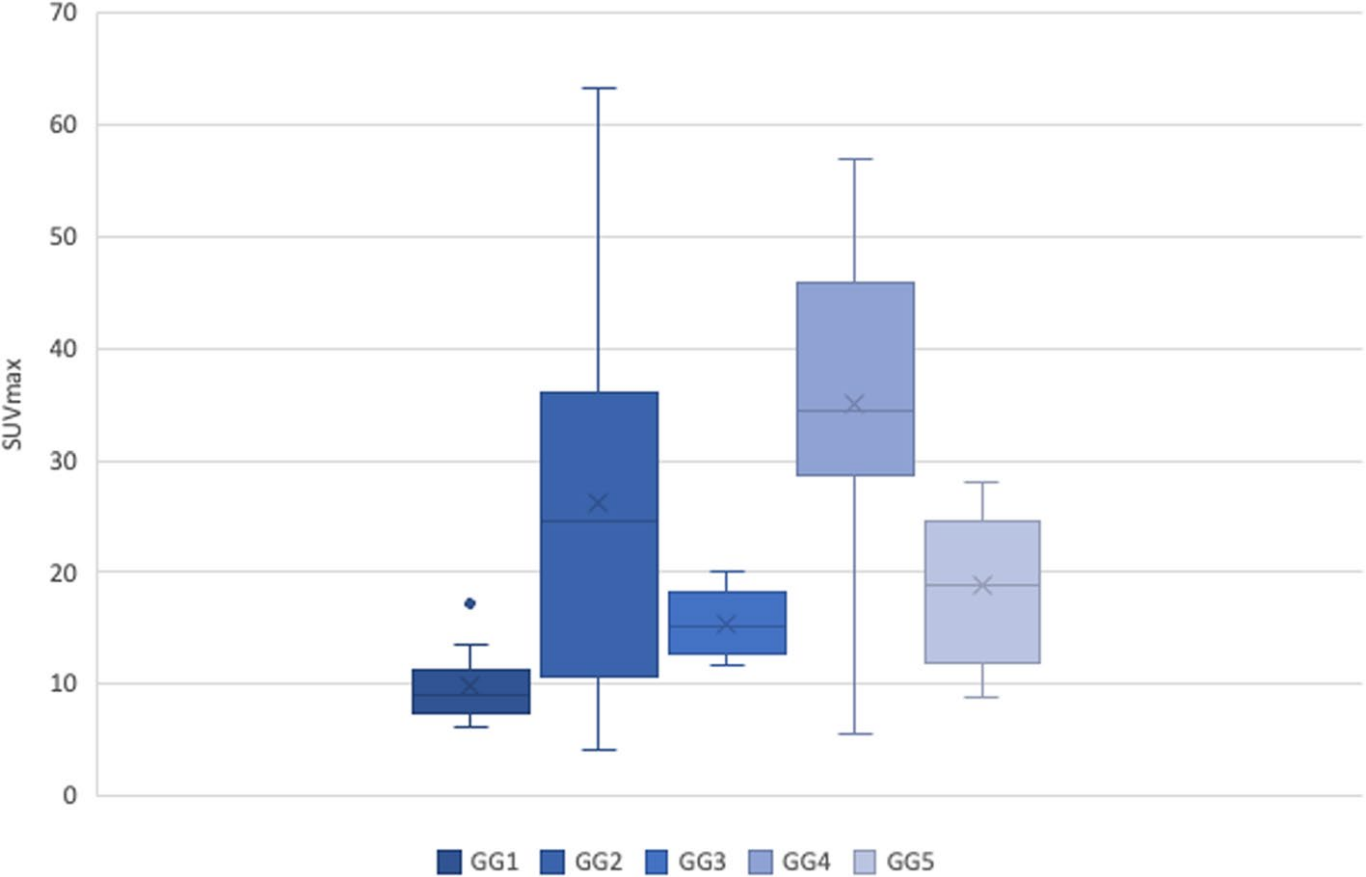
Box and Whisker plot depicting the SUV_max_ across Gleason grade groups

Of the 70 patients who underwent biopsy based on mpMRI and PSMA PET findings, 65 were positive for malignancy, and only five were negative. The histopathological findings of those 65 patients who later underwent prostatectomy confirmed the prostate biopsy results except for one case, which was found to have chronic prostatitis later on histopathological findings. The Gleason score and grade group were calculated based on the histopathological results of the prostatectomy specimen. The Gleason grade group of the patients and their SUV_max_ on PSMA PET is shown in Table 2 and Fig. 2. The mean ± SD SUV_max_ of patients with no malignancy on biopsy was 6.25 ± 1.41.

**Table 2:** Gleason Grade group and Mean SUVmax on PSMA PET-CT scan

Gleason grade group	Mean SUV_max_ (± SD)
1	9.76 (± 4.07)
2	15.50 (± 2.88)
3	18.72 (± 8.03)
4	26.15 (± 16.34)
5	34.99 (± 13.97)

**Fig. 3:**
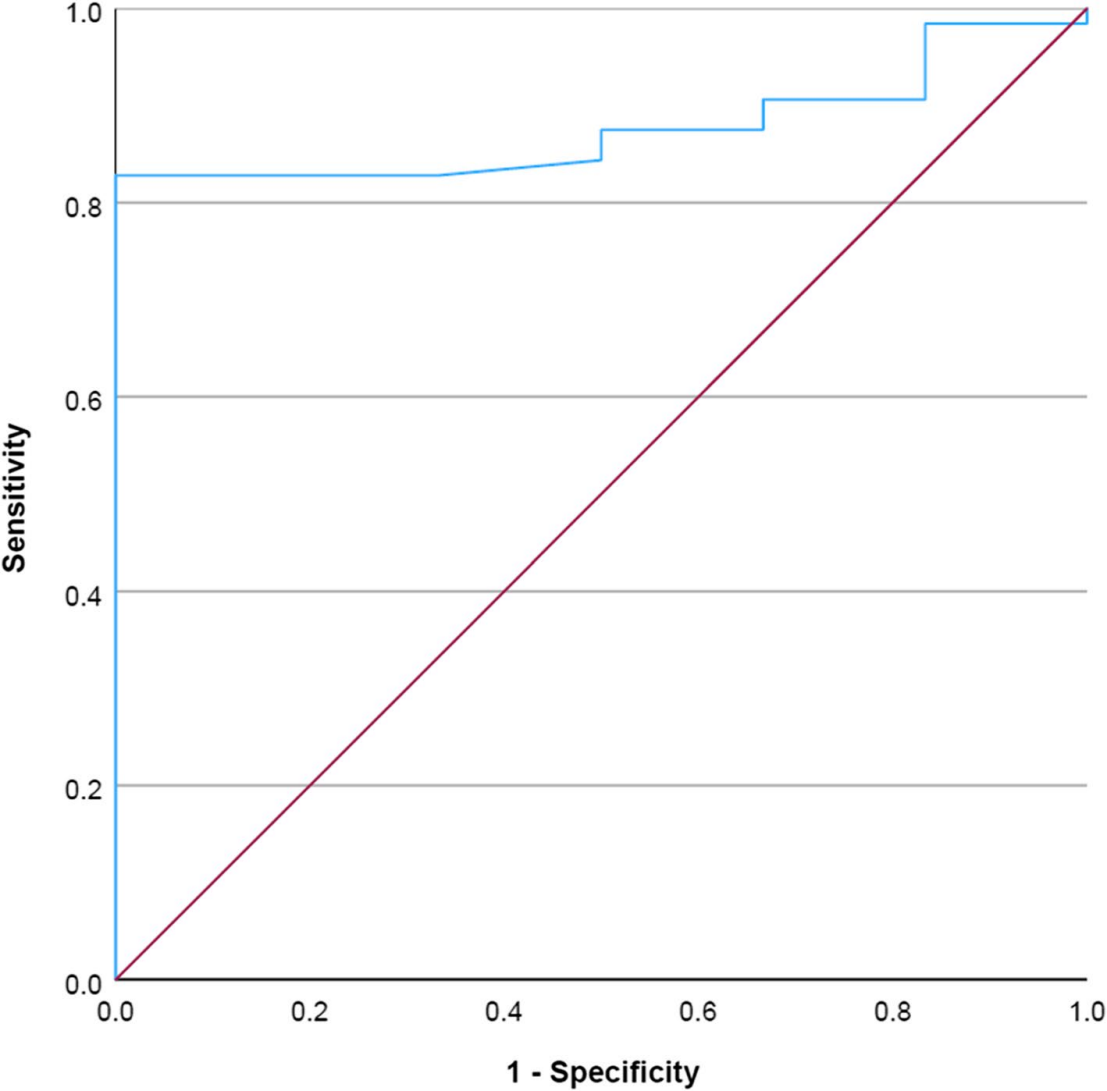
ROC curve depicting diagnostic ability of combined mpMRI and PSMA PET. (Area Under Curve-0.876)

The diagnostic ability of mpMRI (PIRADS 4 and 5 lesions) combined with PSMA PET in accurately predicting malignant lesions has been shown with the help of a ROC curve in Fig. 3.

An AUC of 0.876 depicts a strong positive correlation between the findings of mpMRI and PET with those of the histopathology examination. The value of SUV_max_ at which the results will have maximum sensitivity and specificity was also calculated from the ROC curve. With a sensitivity of 82.8% and specificity of 100%, a SUV_max_ ≥ 8.25 on PSMA PET for a PIRADS 4/5 lesion on mpMRI will help accurately predict malignancy.

We also found that with increasing SUV_max_, the Gleason grade group increased. A significant (*p* < 0.01) Pearson correlation value of 0.735 indicated a strong correlation between SUV_max_ and the Gleason grade group.

## Discussion

Currently, prostate cancer diagnosis relies on suspicion arising from elevated PSA levels and abnormal rectal examinations. In cases where MRI imaging suggests suspicious lesions, a systematic biopsy, with or without a targeted biopsy, may be recommended. PSA density and Free/total PSA ratios are used in cases of equivocal PSA especially in PSA ranging from 4-10 ng/ml. As per the guidelines set forth by the European Association of Urology (EAU), a minimum of 12 cores must be sampled during a prostate biopsy (Guidelines 2022). Nevertheless, due to the potential for adverse effects, many patients express a desire to avoid undergoing a prostate biopsy. Some of these complications encompass sepsis, acute urinary retention, hematuria, hematospermia, prostatitis, epididymitis, persistent pain, dysuria, blood in stools, and an extremely rare 0.1% risk of mortality (Madej et al. 2012; Pinsky et al. 2014). Additionally, patients with contraindications for prostate biopsy, such as those with prostatitis, active urinary tract infections, or those using anticoagulants, inevitably face delays in diagnosis.

The necessity of a prostate biopsy before undergoing radical prostatectomy remains a subject of debate, especially when considering the practices with other solid organ malignancies like kidney and pancreas, where extirpative procedures are often performed without preceding biopsies. Although the European Association of Nuclear Medicine currently supports the use of PSMA imaging in patients with rising PSA after radical treatment to confirm a diagnosis of metachronous oligometastatic disease and PSA persistence following treatment; however, recent results of prospective trials support its use for the initial staging of high-risk patients (Fanti et al. 2021). In our center, we have been transitioning toward a PET targeted transgluteal limited core biopsy approach involving only 2–3 cores, which has demonstrated a significant reduction in complications (Kumar et al. 2022 ). This shift raises questions about the possibility, in select cases, of proceeding with definitive treatment without the need for a biopsy. This unique use has also increased the utilization of PSMA PET-CT for initial diagnosis of localized prostate cancer at our center.

The PRIMARY trial, a prospective multicenter study, explored this idea by suggesting that patients with suspicious PSMA PET and mpMRI findings might be candidates for radical prostatectomy without a prior biopsy (Emmett et al. 2021). Their research indicated that the combination of PSMA PET and mpMRI improved the negative predictive value (NPV) and sensitivity for prostate cancer detection in an MRI triaged population. Meissner et al. conducted a retrospective case series involving 25 patients, concluding that in cases where mpMRI and PSMA PET indicated a high suspicion of prostate cancer, it might be a viable option to forgo a prostate biopsy prior to radical prostatectomy in well-informed, carefully selected patients (Meissner et al. 2022). Chaloupka et al. similarly demonstrated that radical prostatectomy without a preceding biopsy can be safe for diagnosing clinically significant prostate cancer when employing proper preoperative risk stratification, which includes mpMRI and PSMA PET imaging (Chaloupka et al. 1266). Given this context, we undertook a study at our center to assess whether the combined use of PSMA PET and mpMRI can predict the likelihood of cancer and the Gleason grade, aiming to substantiate these findings.

Multi-parametric magnetic resonance imaging (mpMRI) is a comprehensive imaging technique that integrates T2-weighted imaging (T2WI) with diffusion-weighted imaging (DWI), dynamic contrast-enhanced (DCE) perfusion imaging, and spectroscopic imaging (MRSI). This integration aims to enhance the detection, localization, risk assessment, and staging of prostate cancer (Ghai and Haider 2015). Notably, findings from the PRECISION trial highlighted that the MRI-targeted biopsy group identified clinically significant cancer in 38% of cases, in contrast to the 26% detection rate in the standard biopsy group (Kasivisvanathan et al. 2018). While mpMRI effectively reduced the identification of clinically insignificant disease from 22 to 9%, it faced the challenge of a relatively low positive predictive value (PPV) ranging from 34 to 68% (Ghai and Haider 2015; Ahmed et al. 2017). To tackle this limitation associated with mpMRI, researchers have increasingly turned their attention to molecular imaging approaches, such as Ga-68 PSMA PET.

Virtually all types of prostate tissue, including cancerous tissue, contain a type II membrane glycoprotein known as prostate-specific membrane antigen (PSMA). Numerous investigations have demonstrated a direct correlation between PSMA levels and parameters such as Gleason score, T stage, tumor size, and initial PSA levels (Chang xxxx). This inclination of PSMA to indicate the aggressiveness of the tumor can prove advantageous in sparing patients with low-grade cancer from unnecessary treatment. For individuals who are averse to undergoing a biopsy, this predictive capability of PSMA is particularly valuable, as the patient can be directly offered active surveillance.

**Fig. 4:**
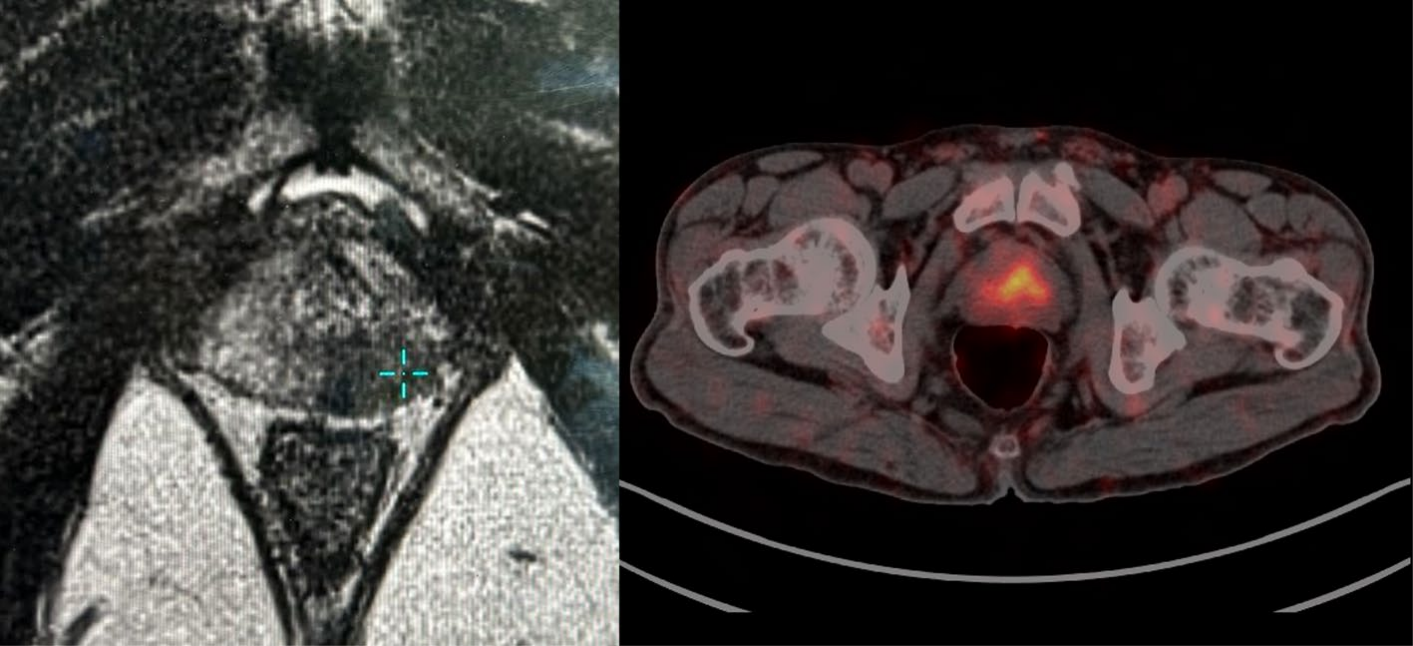
Patient with PIRADS 4 lesion on multi-parametric MRI showing focal tracer uptake is noted in the central zone of the left mid prostate (SUVmax 6.8). Mild inhomogeneous tracer uptake is noted in rest of the prostate gland. Biopsy revealed adenocarcinoma Gleason 3 + 4

**Fig. 5:**
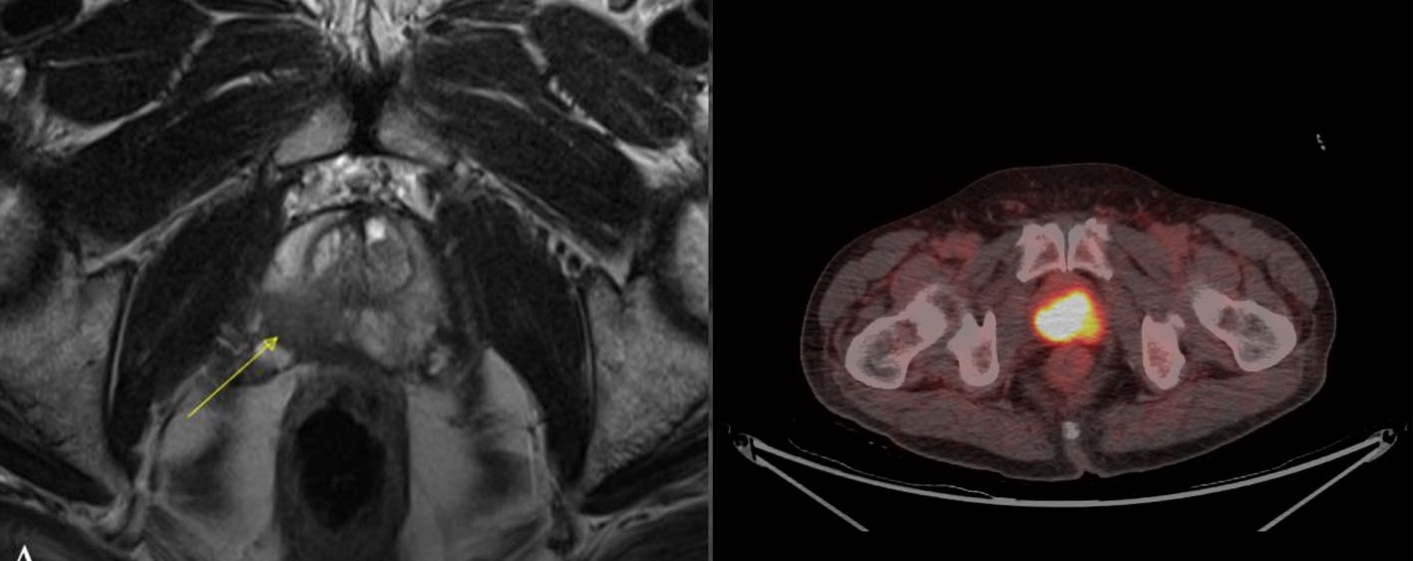
Patient with mpMRI showing PIRADS 5 lesion with intense tracer uptake in the heterogeneously enhancing mass involving the entire prostate gland from base to apex (SUVmax 35.1). Biopsy revealed adenocarcinoma Gleason grade 3 + 4

In this study, we discovered that a PSMA PET SUVmax value ≥ 8.25, in conjunction with a PIRADS score ≥ 4, demonstrated significant accuracy in predicting the presence of significant prostate cancer, with a sensitivity of 82.8% and specificity of 100%. These findings align with those of the PRIMARY trial, which established that a SUVmax exceeding 9 indicated clinically significant prostate cancer (Emmett et al. 2021). This suggests that some men may have the potential to forgo the confirmatory biopsy before proceeding with definitive therapy. Such an approach could lead to a reduction in healthcare costs associated with biopsy procedures and decrease the time from diagnosis to standard treatment. Moreover, it opens the door to establishing a biopsy-free diagnostic pathway based on the results of this study and similar research. This approach would alleviate the complications associated with biopsies and the emotional distress patients experience while awaiting surgery due to biopsy-related delays. Additionally, it creates opportunities for machine learning models to determine the presence or absence of cancer based on MRI and PSMA images. However, challenges related to the cost of dual imaging and false positives must be addressed before implementing a biopsy-free approach for radical prostatectomy. Even in our study, one patient who underwent a biopsy was ultimately diagnosed with chronic prostatitis. Therefore, subjecting patients to the morbidity of radical prostatectomy and its associated complications, including deep vein thrombosis, blood transfusion, urinary incontinence, and erectile dysfunction, remains a contentious issue that requires further consideration and resolution. Also, patients with high PIRADS score can have low or high SUVmax values and hence low SUVmax in a setting of high PIRADS score should be taken with a pinch of salt and should trigger biopsy (Figs. 4 and 5).

MJ Roberts et al*.,* in their study in 2021, concluded that ^68^ Ga-PSMA PET intensity predicts the tumor’s aggressiveness in the form of a higher Gleason grade group on histopathology (Roberts et al. 2020). Our study also confirmed a significantly positive correlation between increasing SUV_max_ and Gleason grade group. Patients with Gleason grade 1 on histopathology had a mean SUV_max_ of 9.76 (± 4.07) on PSMA PET. Patients with Gleason grade group 5 had a mean SUV_max_ of 34.99 (± 13.97). This data can help predict progression-free survival. Also, an increase in SUV_max_ on follow-up may indicate tumor upgrading. This information signifies the need for a reporting guideline for the PSMA PET, similar to PIRADS scoring for mpMRI. With reduction in cores using PSMA PET targeted biopsy, as mentioned earlier we are looking further whether we can offer radical prostatectomy within 2 weeks from biopsy (Kumar et al. 2022). Whether we could completely do away with the biopsy would be the next burning query to be answered.

The outcomes of this investigation yielded promising results, indicating that individuals exhibiting a strong suspicion of prostate carcinoma based on mpMRI and PSMA PET findings (PIRADS ≥ 4 and SUVmax > 8.25) may potentially forego a prostate biopsy before pursuing definitive treatments such as radical prostatectomy. Recent studies have also explored the feasibility of this approach (Meissner et al. 2022; Chaloupka et al. 1266). Nevertheless, it is imperative to counsel patients regarding the slight yet noteworthy risk of a false-positive outcome, which could lead to unnecessary surgical procedures. Additionally, the substantial cost associated with undergoing PET and MRI scans poses a significant challenge, particularly in developing nations, before these methods can be incorporated into routine practice.

The limitations of the study include a small sample size, an inherently high-risk cohort (PIRADS 4 & 5), less number of patients with negative biopsy and low SUVmax. This could possibly be due to referral bias in our setup (Singh et al. 2020). However, this study adds strong value toward establishment of a biopsy-free pathway in patients with localized prostate cancer undergoing two supplementary imaging modalities and may lead to avoidance of biopsy in such patients. Future study in prospective design format and including PIRADS 3 lesions on mpMRI will be carried out our centers to validate the SUVmax cut offs.

## Conclusions

Our results conclude that a combination of PIRADS 4/5 lesion on mpMRI and PSMA positive lesion having SUVmax ≥ 8.25 is highly accurate in predicting prostate cancer. Further studies with a large sample size are needed to quantify the use of SUVmax cutoff values for diagnosing prostate cancer.

## Data Availability

The datasets generated during and/or analyzed during the current study are available from the corresponding author on reasonable request.
